# A systems biology approach to analyse amplification in the JAK2-STAT5 signalling pathway

**DOI:** 10.1186/1752-0509-2-38

**Published:** 2008-04-25

**Authors:** Julio Vera, Julie Bachmann, Andrea C Pfeifer, Verena Becker, Jose A Hormiga, Nestor V Torres Darias, Jens Timmer, Ursula Klingmüller, Olaf Wolkenhauer

**Affiliations:** 1 Systems Biology and Bioinformatics Group, Department of Computer Science, University of Rostock. Rostock, Germany; 2 Systems Biology of Signal Transduction Group. German Cancer Research Center (DKFZ), Heidelberg, Germany; 3 Biochemical Technology Group, Department of Biochemistry and Molecular Biology. University of La Laguna. La Laguna, Spain; 4 Physics Institute, University of Freiburg, Freiburg, Germany

## Abstract

**Background:**

The amplification of signals, defined as an increase in the intensity of a signal through networks of intracellular reactions, is considered one of the essential properties in many cell signalling pathways. Despite of the apparent importance of signal amplification, there have been few attempts to formalise this concept.

**Results:**

In this work we investigate the amplification and responsiveness of the JAK2-STAT5 pathway using a kinetic model. The recruitment of EpoR to the plasma membrane, activation by Epo, and deactivation of the EpoR/JAK2 complex are considered as well as the activation and nucleocytoplasmic shuttling of STAT5. Using qualitative biological knowledge, we first establish the structure of a general power-law model. We then generate a family of models from which we select suitable candidates. The parameter values of the model are estimated from experimental quantitative time-course data. The final model, whether it is conventional model with fixed predefined integer kinetic orders or a model with variable non-integer kinetic orders, is selected on the basis of a good agreement between simulations and the experimental data. The model is used to analyse the responsiveness and amplification properties of the pathway with sustained, transient, and oscillatory stimulation.

**Conclusion:**

The selected kinetic model predicts that the system acts as an amplifier with maximum amplification and sensitivity for input signals whose intensity match physiological values for Epo concentration and with duration in the range of one to 100 minutes. The response of the system reaches saturation for more intense and longer stimulation with Epo. We hypothesise that these properties of the system directly relate to the saturation of Epo receptor activation, its low recruitment to the plasma membrane and intense deactivation as predicted by the model.

## Background

Cellular signal transduction is accomplished by networks of interacting proteins that detect, modulate and transfer cellular signals which control gene expression. A prime example of this is tumour progression and certain oncogenic processes, which directly relate to dysfunctions in signal transduction networks [1,2]. So far the use of mathematical modelling in cell signalling has been limited by the availability of suitable experimental data. However, the systematic development of experimental techniques enabling the generation of time-resolved quantitative data [3-5] facilitates the identification of dynamic pathway models and their parameter values by fitting them to experimental time course data.

The amplification of signals is considered one of the essential properties in most of the cell signalling pathways [6]. The notion of amplification as an increase in the intensity of the signal through the signalling cascade is generally accepted and used to characterise such systems. Surprisingly, there is little work in which a formal definition of amplification is proposed and used together with mathematical models to analyse signalling systems [7-10].

**Figure 1:**
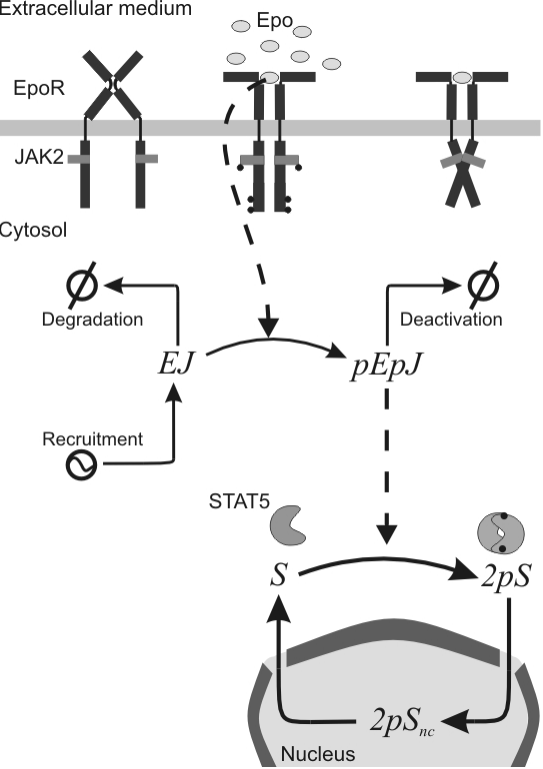
**Structure of the JAK2-STAT5 pathway model proposed**. Legend: *Epo*: concentration of Epo in the extracellular medium; *EJ*: fraction of non-activated EpoR/JAK2 complex; *pEpJ*: fraction of activated EpoR/JAK2 complex; *S*: fraction of non-activated and non-dimerised STAT5 in the cytosol; *DpS*: fraction of activated and dimerised STAT5 in the cytosol; *DpS*_*nc*_: fraction of activated and dimerised STAT5 inside the nucleus.

The Janus kinase – signal transducer and activator of transcription (JAK-STAT) pathways are one of the best-studied cell signalling pathways [11,12]. The JAK2-STAT5 pathway is activated through various receptors, including the erythropoietin receptor (EpoR). Cytokine-activated phosphorylation of EpoR is mediated by the cytosolic kinase JAK2 which is associated with the cytoplasmic domain of EpoR. Upon binding of the hormone erythropoietin (Epo), JAK2 is activated and phosphorylates several tyrosine residues within the cytoplasmic domain of EpoR [13]. Subsequently, the transcription factor STAT5 is recruited to the activated receptor, becomes phosphorylated and thereby gets activated. Upon activation STAT5 homodimerises and migrates to the nucleus, where it initiates the transcription of target genes (see Figure 1 for a simplified representation of this pathway).

Previous work on data-based mathematical modelling of the core module of the JAK2-STAT5 signalling pathway [14] revealed nucleocytoplasmic cycling as an essential building principle of this pathway to closely couple gene transcription to receptor activation. Sensitivity analysis of the model showed that, surprisingly, not the first step of the pathway, i.e. phosphorylation of STAT5 at the receptor, but the parameters describing the shuttling through the nucleus have the major influence on transcription. The prediction of the outcome of an independent experiment based on this theoretical finding could be confirmed. Analysis of the model variables, i.e. unphosphorylated and phosphorylated monomeric STAT5 in the cytoplasm, and dimeric STAT5 in the cytoplasm and the nucleus, demonstrated that in a first round of activation nearly all accessible STAT5 molecules are phosphorylated. Thereby, it was shown that nucleocytoplasmic shuttling serves as a recycling step for the limited pool of STAT5 molecules, thus identifying an implementation of the strategies used by the cell to save energy and resources. The present paper extends this previous work by including a simplified description of the dynamics of EpoR at the plasma membrane (recruitment, degradation, and deactivation) and by analysing amplification and responsiveness of the pathway.

As an alternative to models that are derived on the basis of conventional mass action kinetics with predefined fixed integer kinetic orders, we use a more general power-law formulation that allows for non-integer kinetic orders [15] with the following structure:

(1) $$\begin{array}{cc} \frac{d}{dt}X_{i}=\sum_{j}c_{ij}\cdot \gamma_{j}\cdot \prod_{k=1}^{p}X_{k}^{g_{jk}} & i=1,...,n_{d} \end{array}$$

Where *X*_*i *_represents any of the *n*_*d *_dependent variables of the model (e.g., proteins or phosphoprotein concentrations or levels of gene expression). Here, the biochemical rate *j *is expanded as a product of a rate constant (*γ*_*j*_) and the *p *variables of the system to characteristic kinetic orders (*g*_*jk*_), while *c*_*ij *_are the stoichiometric coefficients of the system describing mass conservation. The main difference between power-law models and conventional ODEs models used in systems biology is that kinetic orders can have non-integer values. There are two main reasons to allow non-integer kinetic orders: firstly, reactions in non-homogenous environments lead to non-integer kinetic orders [16-18] and secondly in the absence of data on the detailed reaction mechanisms one is often forced to condense several steps into simplified representations. This aggregation of information is conveniently represented by power-law expressions, although other alternatives are possible [19,20]. In power-law models, the kinetic orders are parameters of the model and must be estimated from experimental data. Negative values for the kinetic order represent inhibition, while a zero indicates that the variable does not affect the described process. When positive values are considered for a kinetic order, several alternatives are possible: values between zero and one represent a saturation-like behaviour for the rate modelled, and with values higher than one the rate equation models cooperative processes. A kinetic order equal to one means that the system behaves like conventional mass-action kinetic model. By allowing non-integer, positive or negative, kinetic orders we consider for the same model structure a larger class of kinetic models from which we can select a suitable candidate.

However, the increased generality comes at a price when it comes to the estimation of parameter values from experimental data for larger networks. While for conventional models the kinetic orders are decided *a priori*, in power-law representations the number of parameters that must be extracted from data is therefore larger. The identifiability problem means that there could be multiple sets of parameter values for which the model fits the data equally well. Analytical approaches for the inference of identifiability are limited to rather low dimensional systems [21], while approaches that are applicable to large systems [22,23] rely on linearisation that question their reliability. Recent results based on non-parametric bootstrap-based approaches [24] will allow for reliable identifiability analysis also for high dimensional systems.

Next, we discuss the structure and equations of the mathematical model proposed and the process by which parameter values were estimated. In addition, we performed a model-based analysis of the responsiveness and amplification of the system, for which we propose a formal definition of these variables. The experimental and computational methods used to set up the model for the JAK2-STAT5 pathway are summarised at the end of the manuscript.

## Results and Discussion

### Mathematical Model and Parameter Estimation

In our model, EpoR and JAK2 were assumed to form a stable complex, EpoR/JAK2, for all biochemical processes included. All variables in the model describing the considered states of EpoR refer to populations of the receptor at the plasma membrane. Based on experimental data obtained during our investigation (data not shown), we assume in our model that the dynamics of the intermediate state of the receptor in which only JAK2 is activated is negligible in the description of the system. Thus, two possible states for the EpoR/JAK2 complex were considered: i) EpoR/JAK2 not bound to Epo, *EJ*; and ii) activated Epo-bound EpoR/JAK2 complex, *pEpJ*. The receptor activation was modelled with a term depending on the Epo concentration and the amount of non-activated receptor complex at the plasma membrane $\left(\gamma_{2}\cdot EJ^{g_{1}}\cdot Epo^{g_{2}}\right)$ A rate equation accounting for the degradation of activated Epo receptor was also included in the model. Since the *k*_*off *_of the Epo binding to the human EpoR is much smaller than the *k*_*on *_[25], we assumed that effects of the release and reactivation of the related murine EpoR can be neglected, which was also tested in preliminary models. Actually, initial explorations considering the reactivation of EpoR were not consistent with the experimental data used in this work (data not shown). The structure of the model was completed by the inclusion of terms that describe the recruitment of new EpoR/JAK2 complexes to the plasma membrane and the degradation of non-activated EpoR/JAK2 complexes.

Three possible states have been considered for STAT5 in the model: i) non-activated and monomeric STAT5 in the cytosol, *S*; ii) activated STAT5 in the cytosol, *DpS*; and iii) activated STAT5 in the nucleus, *DpS_nc_*. STAT5 activation comes through phosphorylation and dimerisation. Our preliminary results suggested that dimerisation is a fast processs and therefore the slower phosphorylation of STAT5 in the receptor complex leads the activation process. Thus, model reduction was applied and the aggregation of both processes was then represented with a unique rate equation ($\gamma_{5}\cdot S^{g_{6}}\cdot pEpJ^{g_{7}}$, see Additional file 1). Since the dimerisation of STAT5 is considered a fast process, the model assumes that the protein dimerises immediately after monomeric phosphorylation and the variable that describes monomeric phosphorylated STAT5 is neglected. We note that STAT5 can only be considered activated after the dimerisation of two phosphorylated monomers. Experimental data describing the translocation of activated STAT5 to the nucleus and its dynamical behaviour inside the nucleus are currently not available, and therefore the differential equations describing such processes have not been formulated in detail. Thus, we model the fraction of STAT5 inside the nucleus with a single state variable. The processes considered in the model are the activation of STAT5 by the activated receptor complex EpoR/JAK2, the translocation of cytosolic activated STAT5 to the nucleus, and the deactivation and subsequent translocation of nuclear STAT5 back to the cytoplasm. In accordance with [14], only nuclear activated STAT5 can be deactivated in our model. The total amount of STAT5 is supposed to be constant.

Finally, the concentration of Epo in the extracellular medium, *Epo*, is considered the input signal of the system. The resulting model has five dependent variables and one input variable. Figure 1 illustrates the structure of the model proposed. Only the states of the proteins and the processes that have been discussed are included. The differential equations for the model were formulated using power-law terms:

(3.1) $$\frac{d}{dt}EJ=\gamma_{1}-\gamma_{2}\cdot EJ^{g_{1}}\cdot Epo^{g_{2}}-\gamma_{1}\cdot EJ^{g_{3}}$$

(3.2) $$\frac{d}{dt}pEpJ=\gamma_{2}\cdot EJ^{g_{1}}\cdot Epo^{g_{2}}-\gamma_{3}\cdot pEpJ^{g_{4}}$$

(3.3) $$\frac{d}{dt}S=2\cdot \gamma_{4}\cdot {\left(DpS_{nc}\left(t-\tau \right)\right)}^{g_{5}}-2\cdot \gamma_{5}\cdot S^{g_{6}}\cdot pEpJ^{g_{7}}$$

(3.4) $$\frac{d}{dt}DpS=\gamma_{5}\cdot S^{g_{6}}\cdot pEpJ^{g_{7}}-\gamma_{6}\cdot DpS^{g_{8}}$$

(3.5) $$\frac{d}{dt}DpS_{nc}=\gamma_{6}\cdot DpS^{g_{8}}-\gamma_{4}\cdot {\left(DpS_{nc}\left(t-\tau \right)\right)}^{g_{5}}$$

In this model we assume that, after the long period of starvation before starting the experiment, the system reaches a steady-state level of EpoR at the plasma membrane. Under these conditions and in the absence of stimulation, there is equilibrium between receptor recruitment and degradation, which makes the rate constants in both terms (recruitment rate and first order degradation for the receptor) equal for the normalised variables used in the model. The effect of the dimerisation process was also considered in the formulation of the equations, which leads to stoichiometric coefficients of value two in Equation (3.3). Following the ideas proposed in [14], a possible delay, *τ*, was included in the rate that describes the deactivation of *DpS_nc _*in the nucleus and subsequent translocation to the cytosol.

The experimental data available were converted into the normalised scale [5] defined in Sup. Mat. A1 [see Additional file 1]. Data from two replicate experiments were available and used as independent experiments for parameter estimation. In these experiments, the cytoplasmic concentration of activated STAT5, [pSTAT5_cyt_], and the concentration of activated EpoR/JAK2 complex, [pEpoR], were measured in several time points. Additionally, the extracellular concentration of Epo, [Epo], was measured in an independent equivalent experiment. Additional algebraic equations, reflecting the relation between the measured quantities (observables) and the variables, were defined in the model:

(4) [Epo] = *Epo *[pEpoR] = *pEpJ *[pSTAT5_cyt_] = 2·*DpS*

The variables on the left-hand side represent the observables, while the right-hand side represent the variables considered in the model. Further details on data processing are discused in Sup. Mat. A2 [see Additional file 1]. Several models were tested, including models with fixed predefined integer kinetic orders, with and without time delay in the STAT5 cycling, and several models with an increasing number of variable non-integer kinetic orders. In this latter case, variable non-integer kinetic orders related to terms representing a higher simplification of the dynamics were tested first (EpoR and STAT5 activation) and after that, the number of terms with non-integer kinetic order was systematically increased. The best compromise between an appropriate data fitting and a suitable number of parameters to be estimated was a model with fixed integer kinetic orders and no explicit time-delay for *DpS_nc _*deactivation. Note, that although the chosen model here has no time delay as in [14], there is no contradiction between the two models. We do not represent the time delay implicitly but here we have a new state variable *DpS_nc _*representing the fraction of activated STAT5 in the nucleus. The power-law model with non-integer kinetic orders for the term describing EpoR/JAK2 complex activation fitted the available data better (the objective function, *F_obj_*, is 20% smaller than in the kinetic model), but the limited improvement obtained did not justify the choice of this model and we therefore followed the parsimony principle and selected the simpler and yet satisfactory model with fixed predefined integer kinetic orders. The procedure used and the different options analysed are discussed in Sup. Mat. A2 [see Additional file 1]. In all cases, parameters were computed for biologically relevant intervals in the parameter space using a genetic algorithm. The parameter values for the chosen model as well as the initial conditions used for parameter estimation are summarised in Table 1.

**Table 1:** Values of the parameters in the selected solution

**Parameter**	*g_1 _*- *g_8 _*		*τ*
Value	1		0
**Parameter**	*γ*_1_	*γ*_2_	*γ*_3_
Value	0.0025 (± 0.0004)	0.2531(± 0.0006)	0.0175 (± 0.0092)
**Parameter**	*γ*_4_	*γ*_5_	*γ*_6_
Value	0.4674 (± 0.0136)	0.3631 (± 0.0102)	0.454 (± 0.0151)
**Parameter**	*Epo(t = 0)*	*EJ(t = 0)*	*pEpJ(t = 0)*
Value	1	1	0
**Parameter**	*S(t = 0)*	*DpS(t = 0)*	*DpS*_*nc*_*(t = 0)*
Value	1	0	0

**Figure 2:**
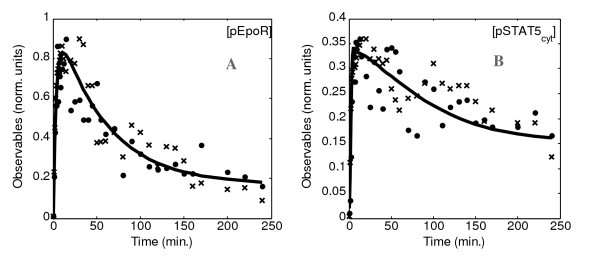
**Data fitting of the selected solution for the observables**. A: activated EpoR, [pEpoR]. B: activated cytosolic STAT5, [pSTAT5_cyt_]. Data from two replicates were used as independent experiments. The quantitative data obtained from the two experiments (data points represented as crosses for experiment 1 and points for experiment 2) are compared with the solution of the model identification process (lines). Experimental data were obtained from BaF3-EpoR cells (proB cells exogenously expressing the murine EpoR) stimulated with 5 units/ml Epo and normalised as defined in Sup. Mat. A1 [see Additional file 1].

The model trajectories obtained for the chosen solution are depicted in Figure 2. The general behaviour of the system is reproduced despite the fact that the differences between the data obtained in the two replicates of the experiment are significant for some time points. We notice that the dynamics of phosphorylated EpoR is much better delimited by the experimental data and therefore the fit of the data is much clearer. The fit is also quantified with the objective function described in Equation 2 in Table A3 in Sup. Mat. A2 [see Additional file 1].

### Analysis of Amplification

To analyse how the signal is amplified through the pathway, we define the logarithmic amplification factor (*LA*) between two activated intermediates in a signalling pathway X* and Y* (with Y* downstream in the pathway) with the following equation:

(6) $$LA=\log\left(\frac{\int_{0}^{T}V_{Y^{*}}^{+}\left(t\right)dt}{\int_{0}^{T}V_{X^{*}}^{+}\left(t\right)dt}\right)$$

Where *T *is the duration of stimulation, *LA *is the logarithm of the ratio between the total productions of both intermediates during the signalling process considered. The total production of an intermediate is described by the integral of the net activation rate during the stimulation process. Considering this definition, a system amplifies between two steps in the pathway when *LA *is higher than zero. If *LA *is smaller than zero, the system provokes an attenuation of the signal. In the scale proposed, a value of one for *LA *implies that on average each molecule of X* produces ten molecules of Y*, while a value of minus one represents that ten molecules of X* produce on average one molecule of Y*. In Table 2 we propose a classification in significant intervals for the value of *LA *in terms of the ability of the system to amplify.

**Table 2:** Classification for the values of logarithmic amplification (*LA) *in significant intervals

*LA *< -1	Strong attenuation
-1 <*LA *< 0	Attenuation
0 <*LA *< 1	Amplification
*LA *> 1	Strong amplification

**Figure 3:**
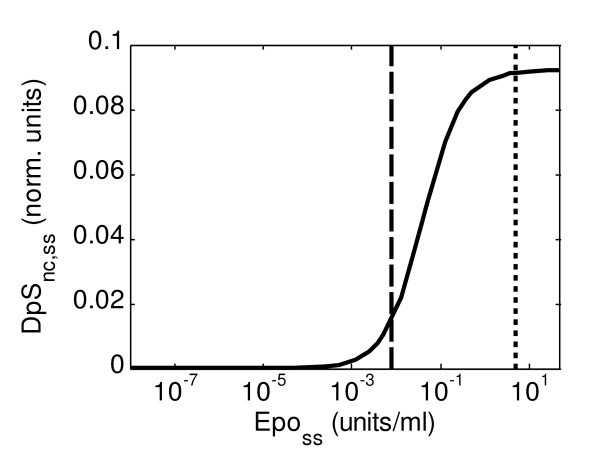
**Steady-state values of *DpS*_*nc *_(*DpS*_*nc*,*ss*_) for different values of sustained stimulation on Epo (*Epo*_*ss*_)**. The dashed black line indicates the physiological value for serum concentration of Epo (aprox. 7.9·10^-3 ^units/ml), while the finely dashed line indicates the concentration of Epo used in the experiments performed (5 units/ml).

In order to analyse the responsiveness and the ability of the system to amplify signals, the performance of the system was analysed via mathematical simulation assuming three different conditions: sustained stimulation, transient stimulation and oscillatory stimulation by Epo. In case of a sustained stimulus, we analysed the response of the system in terms of the steady-state induced in the system for different values of constant Epo concentration in the extracellular medium, *Epo*_*ss*_, from very low concentrations, *Epo*_*ss *_= 10^-8 ^units/ml, to concentrations up to tenfold the initial concentration used in the experiments performed, *Epo*_*ss *_= 50.0 units/ml. Under normal conditions, the physiological serum concentration of Epo in mice is 7.9·10^-3 ^units/ml [26]. We have computed the steady-state values of *DpS*_*nc *_and they are shown in Figure 3. The logarithmic scale was used for the values of *Epo*_*ss*_.

The figure shows clearly a sigmoidal behaviour in the log scale of *Epo*_*ss*_. We can see that the system maximally responds to changes in concentration of Epo in the interval 10^-3^-10^-1^, which includes the normal range of Epo concentration in mouse serum (the behaviour is similar in case of *pEpJ *[see Additional file 1]). For smaller sustained stimuli the system is not significantly activated, while for intense stimuli the system reaches saturation and becomes virtually insensitive to any increase in the stimulus. Similar behaviour has been previously described as a typical feature of amplifying signal transduction pathways [7]. We analysed also the logarithmic amplification factor, *LA*, between the activated receptor, *pEpJ*, and the activated dimerised STAT5 in the nucleus, *DpS*_*nc*_. The total production in steady-state is described by the integral of the net activation rate of the considered intermediate. If we use the suggested definition in this case, we obtain the following:

(7.1) $$LA=\log\left(R_{STAT5/EJ}\frac{\int_{0}^{T}V_{DpS_{nc}}^{+}\left(t\right)\cdot dt}{\int_{0}^{T}V_{pEpJ}^{+}\left(t\right)\cdot dt}\right)$$

(7.2) $$LA=\log\left(R_{STAT5/EJ}\frac{V_{DpS_{nc}}^{+SS}}{V_{pEpJ}^{+SS}}\right)$$

(7.3) $$\begin{array}{cc} V_{pEpJ}^{+}=\gamma_{2}\cdot EJ\cdot Epo & V_{DpS_{nc}}^{+}=\gamma_{5}\cdot S\cdot pEpJ \end{array}$$

where $V_{pEpJ}^{+SS}$ is the steady-state value of $V_{pEpJ}^{+}$ and $V_{DpS_{nc}}^{+SS}$ is the steady-state value of $V_{DpS_{nc}}^{+}$ for the considered sustained concentration of *Epo*. *R*_*STAT5/EJ *_is the ratio between the total amount of STAT5 and total amount of the EpoR at the cell surface. Since in our model we use normalised units, this ratio must be calculated in order to quantify the real value of amplification. The preliminary estimates for this ratio in the investigated cell line assigns a value around *R*_*STAT5/EJ *_= 10 (data not shown). The inclusion of this factor is useful in order to quantify the numerical value of the amplification (it is acting as a translation factor in the *LA*-axis), but the rest of properties of the *LA *function (shape of the curve, scale, position of maxima and minima) depend on the intrinsic properties of the model and are not affected by the value of this ratio (*LA *is a logarithmic property; further explanations in Sup. Mat. A3 [see Additional file 1]). The definition in Equation (7.1) is valid for any kind of stimulus applied to the system (sustained, transient or oscillatory), while Equation (7.2) is the specific formulation for a sustained stimulus.

**Figure 4:**
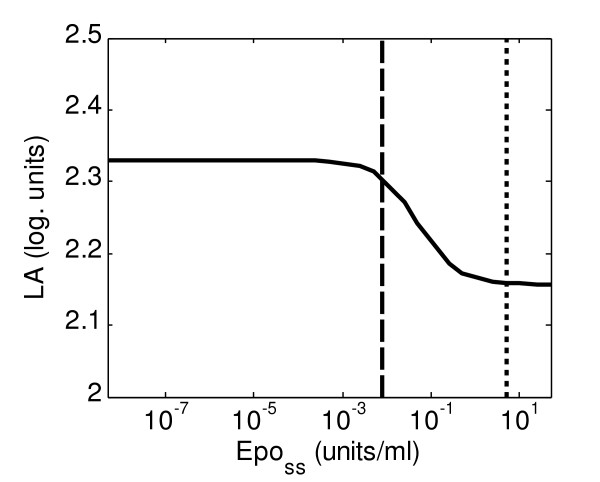
**Logarithmic amplification, *LA *(log. units, see Equation 7.1), for different values of sustained stimulation with Epo (*Epo*_*ss*_)**. *LA *measures the signal amplification between *pEpJ *and *DpS*_*nc*_. The dashed black line indicates the physiological value for serum concentration of Epo (aprox. 7.9·10^-3 ^units/ml), while the finely dashed line indicates the concentration of Epo used in the experiments performed (5 units/ml).

Figure 4 shows the results obtained for the considered interval of sustained stimulus. The logarithmic amplification factor has a value slightly higher than two (*LA *= 2) for all different values of sustained stimulation simulated, which implies an intense amplification of the signal. Thus, our model predicts that an activated receptor can on average activate and induce the nuclear translocation of up to 125 units of STAT5 before its deactivation. In addition, the maximal increase in amplification is in the interval 10^-4^-10^-1^; smaller stimuli produce a higher amplification, which implies that the system reacts sensitively to weak stimulation.

In case of a transient stimulus, the stimulation by Epo was characterised by two properties: the average duration of the stimulus, *T*_*Epo*_, and the average value of the Epo concentration during transient stimulation, *Epo*_*tr*_. In both cases we adapted the definitions proposed in [8] (a complete description is available in Sup. Mat. A4 [see Additional file 1]). Moreover, the variables considered for the analysis of a transient stimulation were the average amount of *pEpJ *(*pEpJ*_*tr*_) and *DpS*_*nc *_(*DpS*_*nc*,*tr*_*) *during the stimulation, which are the transient stimulation "equivalent" for *Epo*_*ss *_*and DpS*_*ss*_. In the simulations, the initial values for the variables were consistent with the initial conditions (representing conditions after serum starvation) used in data fitting (Sup. Mat. A1 [see Additional file 1]). The stimuli analysed had a duration between 0.1 and 10^4 ^minutes and an average stimulus concentration between 10^-6 ^and 500 units/ml:

(8) *T_Epo _*∈ [0.1, 10^4^] min *Epo*_*tr *_∈ [10^-6^,500] units/ml

**Figure 5:**
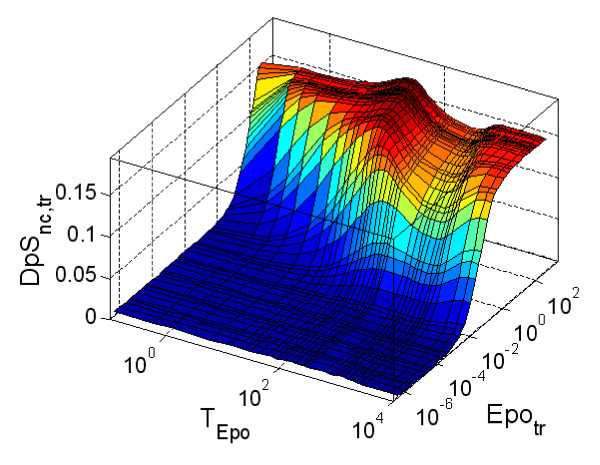
**Average fraction of dimerised phosphorylated STAT5 in the nucleus (*DpS*_*nc,tr*_) (norm. units), during transient stimulation**. The behaviour of the system was analysed for transient stimulation with an average duration of *T*_*Epo *_∈ [0.1, 10^4^] minutes, and an average concentration of *Epo*_*tr *_∈ [10^-6^,500] units/ml.

**Figure 6:**
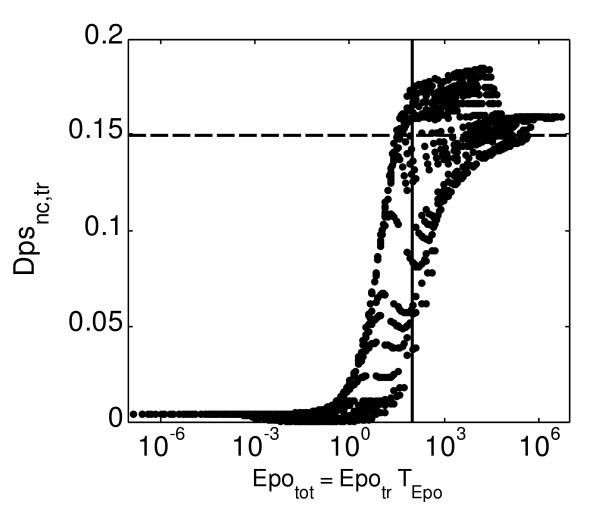
**Average fraction of dimerised phosphorylated STAT5 in the nucleus (*DpS*_*nc,tr*_) (norm. units versus the total amount of Epo (*Epo*_*tot*_) provided during the transient stimulation**. The system was analysed for transient stimulation with total amount of Epo *Epo*_*tot *_∈ [10^-8^,10^6^] units/ml. The solid black line is used to highlight the different values of *DpS*_*nc*,*tr *_obtained for a total amount of Epo of one hundred units. In this case the average of the system response ranges between 0.05 and 0.17 normalised units. The dashed black line is used to highlight how in the interval of intense stimulation input signals with totally different properties (*Epo*_*tot *_∈ [10^1^,10^5^] units/ml) produce output signal with identical average intensity *DpS*_*nc*,*tr*_.

Figure 5 shows the response of the system in *DpS*_*nc*,*tr *_when transient stimulation of the system with the properties stated were simulated and analysed (Sup. Mat. A6 includes the figures on *pEpJ *[see Additional file 1]). The system reaches maximum average activation for stimuli with average intensity, *Epo*_*tr*_, higher than 0.01 and average duration, *T*_*Epo*_, between approximately 10 and 100 minutes. For longer stimulation, even at very high concentrations of Epo, there is a significant loss in *DpS*_*nc*,tr_. For input signal longer than 500 minutes there is a partial recovery of the average signal intensity *DpS*_*nc*,tr _and a plateau finally appears (which is even more apparent for *pEpJ*_*tr *_[see Additional file 1]) for very long stimulation, which means that the system is not able to produce stronger responses for more intense stimuli. We suggest that this behaviour relates to the effect of limited recruitment of new EpoR to the plasma membrane, which is unable of faster recovery after very intense stimulation. We notice that is special features of the figure appear even for other computed solutions (data not shown) and seems to be a structural property of the model. In the range of the Epo pulse duration (*T*_*Epo*_) between 1 and 100 minutes and for average stimulus concentration (*Epo*_*tr*_) around the physiological values, [5.10^-3^, 5.10^-1^], the system shows maximum sensitivity to changes in the properties of the input signal. We furthermore investigated the responsiveness of the system predicted by the model with respect to the total amount of stimulus provided (*Epo*_*tot*_), which in our simulations is represented by the product *Epo*_*tot *_= *Epo*_*tr *_· *T*_*Epo *_(Sup. Mat. A4 contains further explanations [see Additional file 1]). Figure 6 shows our results, which condense the information depicted in Figure 5. The characteristic sigmoid-like behaviour of the model is also visualised in this figure. In addition, when a low stimulus is provided, the average response of the system (represented by *DpS*_*nc*,*tr*_) does not depend on the features of the transient stimulus and stimulation with different duration and average intensity but the same total amount of Epo produces an almost identical response (Figure 6). In contrast, for a larger stimulus the response of the system will depend on the features of the input signal: different signals with the same total amount but different duration or average intensity will produce responses that can differ on average in ± 0.05 normalised units for *DpS*_*n*,*trc*_, that is 50% of the average value (see solid black line in Figure 6). Interestingly, in case of medium and high *Epo*_*tot*_, there is for any value of *DpS*_*nc,tr *_an interval of values for *Epo*_*tot *_in which signals that differ in all their properties will produce the same average output signal.

**Figure 7:**
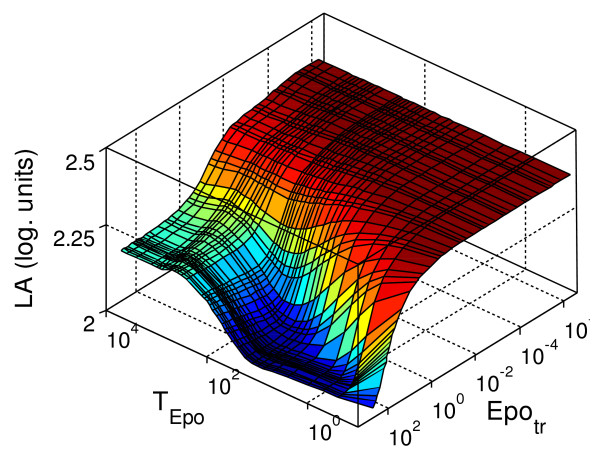
**Logarithmic amplification, *LA *(log. units, see Equation 7.1), measured for different transient stimuli**. *LA *measures the signal amplification between *pEpJ *and *DpS*_*nc*_. The behaviour of the system was analysed for transient stimulation with an average duration of *T*_*Epo *_∈ [0.1, 10^4^] minutes, and an average concentration of *Epo*_*tr *_∈ [10^-6^,500] units/ml.

When we analysed the logarithmic amplification for a transient stimulus (Figure 7), we found that the range of values is again reduced but the average value is higher than two. The minimum amplification is reached for a transient stimulus with intermediate duration and high intensity, while the system shows a higher factor of amplification for short, weak stimulation in accordance with the results obtained for sustained stimulation. The maximum sensitivity of the amplification factor to changes in the signal is in the interval previously determined (*T*_*Epo *_∈ [1,500 min], *Epo*_*tr *_∈ [5.10^-3^, 5.10^-1^])

**Figure 8:**
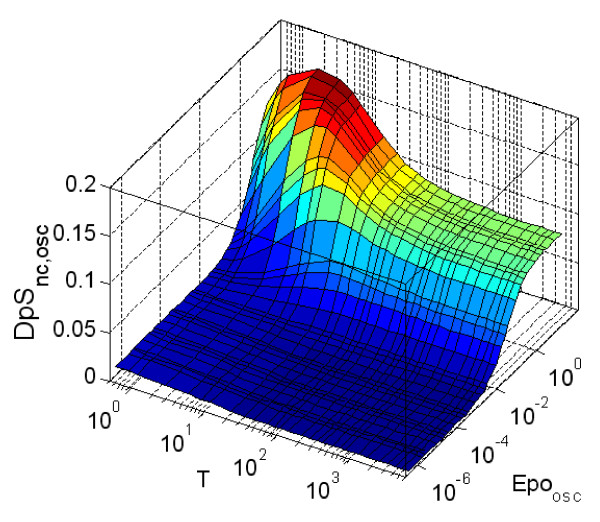
**Average fraction of dimerised phosphorylated STAT5 in the nucleus (*DpS*_*nc*,*osc*_) (norm. units) during oscillatory stimulation**. The simulated data was averaged for several following periods. The behaviour of the system was analysed for oscillatory stimuli with an average period duration T ∈ [0.5, 1440] minutes, and an average concentration of *Epo*_*ocs *_∈ [10^-6^, 10] units/ml. In all the simulations, the signal was averaged for 12 periods of the oscillatory stimulus.

The dynamics of the system with oscillatory Epo concentration were also considered. For simulations, we did not choose perfect sinusoidal oscillatory signals but a design based on truncated triangular signals (Sup. Mat. A5 includes further explanations [see Additional file 1]). In [27] a physiological daily oscillation of the Epo concentration in the blood is described in which Epo is maintained at an almost stable value during daytime but reduces to half its value during the night. Two periods of transition are described that we adopted to describe the oscillatory signals used in our simulations. The oscillations in the input signal were characterised by two properties: the period of the oscillatory signal (T) which is the time between two consecutive maxima, and the average value of Epo during the oscillation (*Epo*_*osc*_). The average value of *pEpJ *(*pEpJ*_*osc*_) and *DpS*_*nc *_(*DpS*_*nc*,*osc*_) during the oscillation were defined and computed (Sup. Mat. A7 [see Additional file 1]). Our analysis suggested that the average of the signals for a number of periods between eight and twelve is sufficient to avoid the transient behaviour from the start of the simulations. We explored the performance of the system for oscillations with a period between half a minute and one day and with *Epo*_*osc *_in the interval studied in the previous cases ([10^-6^,10] units/ml). Figure 8 shows the results obtained for *DpS*_*nc*,*osc *_(Sup. Mat. A7 contains figures for *pEpJ*_*osc *_[see Additional file 1]).

**Figure 9:**
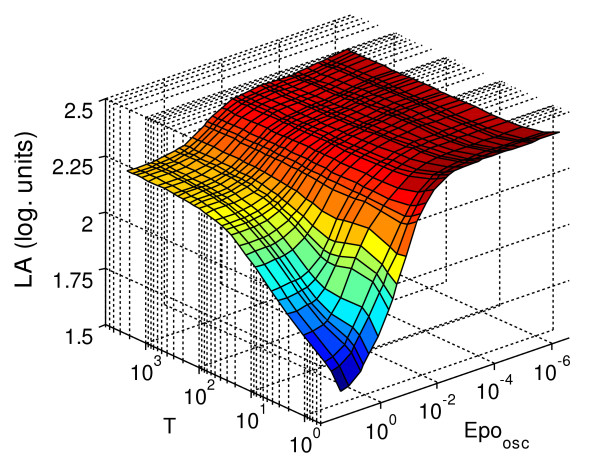
**Logarithmic amplification, *LA *(log units, see Equation 7.1), measured for different oscillatory stimuli**. *LA *measures the signal amplification between *pEpJ *and *DpS*_*nc*_. The simulated data was averaged for several subsequent periods. The behaviour of the system was analysed for oscillatory stimuli with an average period duration T ∈ [0.5, 1440] minutes, and an average concentration of *Epo*_*osc *_∈ [10^-6^, 10] units/ml. In all the simulations, the signal was averaged for 12 periods of the oscillatory stimulus.

In Figure 8, we can see that the maximum response of the system for *DpS_nc _*is reached for oscillatory signals with a period of 5–50 minutes and an average intensity between 0.1 and one (*DpS_nc _*≈ 0.15). This time interval coincides with the time scale suggested in [14] for the nucleocytoplasmic shuttling of STAT5, which could indicate a coupling between the frequency of the oscillations and the maximum performance of the pathway. For intense stimuli with longer period, the system reached a plateau in the average activated STAT5 in the nucleus at a value of half the maximum (*DpS_nc _*≈ 0.075). In case of the logarithmic amplification (Figure 9), a plateau with the highest value of amplification appears for oscillations with periods from short to long (T between 10–1000 min.) and weak stimuli (*Epo_osc _*< 10^–3^). Again, the maximum sensitivity to changes in the properties of the input signal appears for intermediate, physiologically feasible Epo concentration (*Epo_osc_*) and for oscillations with a period T of 5–50 minutes. Intense stimulation with a short period produces a minimum in the amplification of the system, although the system is still strongly activated.

Interestingly, the properties of responsiveness and amplification predicted by the model with variable non-integer kinetic orders are very similar to the ones discussed here (Sup. Mat. A7 contains figures comparing both models [see Additional file 1]). The sigmoid-like curve on the responsiveness (*DpS*_*nc*_) for sustained stimulation appears also for this model but is steeper and appears shifted one order of magnitude to higher values of sustained stimulus (Figure A7.1 [see Additional file 1]). Surprisingly, the logarithmic amplification (*LA*) in both models differs for very small sustained stimulus: the model with fixed predefined integer kinetic orders predicts a small variation on the values of the amplification through the whole interval of Epo values whereas the one with variable non-integer kinetic orders predicts an intense loss of amplification, which becomes negative (signal attenuation) for *Epo *≤ 10^-5 ^units/ml. Our analysis suggests that this feature is shared by the best power-law solutions (first ten of the selected power-law solutions, all them with steep sigmoidal curves). The other power-law solutions have a *LA *curve similar to the one obtained for the selected model (data not shown).

When transient stimulation is analysed, both models predict a similar shape for the surface accounting for the responsiveness and logarithmic gain of the system with respect to the features of the transient stimulation (*Epo*_*tr*_, *T*_*Epo*_), but again the sigmoidal curve for the model with variable non-integer kinetic orders is steeper and shifted (Sup. Mat Figure A7.2 [see Additional file 1]). The behaviour of the chosen model for oscillatory signals reproduces the features of the previously analysed signals (Sup. Mat Figure A7.3 [see Additional file 1]).

Finally, recent studies suggest the time-dependent recruitment of phosphatases like SHP-1 and other negative regulators like SOCS proteins as possible mechanisms to control cytokine responses, in particular in the pathway studied [28]. Any attempt to understand the behaviour of the JAK2-STAT5 pathway in depth requires to integrate the effect of these regulatory proteins in the mathematical model proposed and to generate the adequate experimental data to characterise these effects. The results that we show in this article should be confirmed or refined with this extended (feedback loop-controlled) model, additional experimental data and validation.

## Conclusion

A mathematical model based on ordinary differential equations and represented in power-law terms was developed for the JAK2-STAT5 pathway. Since the data available were rather limited, a strategy to reduce the complexity of the model was formulated following the strategy proposed in [15]. Using the available biological knowledge, several terms in the models were approximated by conventional kinetic terms with kinetic orders equal to one. With the remaining kinetic orders, a strategy of gradually increasing complexity in the structure of the model was used, which allowed a higher number of kinetic orders to be different to one for each iteration. The inclusion of a time delay in the STAT5 cycling was also considered. Although the best numerical fit to the data was obtained for a model with variable non-integer kinetic orders, a compromise between satisfactory reproducibility of experimental data and reduced complexity of the model lead to a simpler model with fixed predefined integer kinetic orders.

The responsiveness and amplification of the system were studied for sustained, transient, and oscillatory stimuli. Regarding responsiveness, we focussed on the value of activated STAT5 in the nucleus (*DpS_nc_*), while the analysis of the fraction of activated receptors has been included in Sup. Mat. A7 [see Additional file 1]. To measure the ability of the system to amplify the signal we defined the logarithmic amplification (*LA*) as the logarithm of the ratio between the total amount of activated STAT5 in the nucleus (*DpS_nc_*) and the activated receptor (*pEpJ*) during the analysed processes. A scale of values was set up in which positive values imply amplification of the signal, while negative values imply attenuation.

For the fraction of activated dimerised STAT5 in the nucleus (*DpS_nc_*) and the logarithmic amplification (*LA*) the system seems especially sensitive in the range of physiological Epo concentrations [26]. Within this range changes in the properties of the stimulus for the three kinds of signals studied (sustained, transient, and oscillatory) provoke significant changes in the response of the system. For a stronger stimulus the system reaches saturation, while for a weaker stimulus there is no significant response (the system stays almost switched off). This could imply that the system is designed to have the maximum sensitivity to the intensity of the stimuli within the biologically feasible interval. The highest sensitivity of the system is not reached for transient stimulation or oscillatory input signals with very long duration (higher than one hundred minutes in both cases) but for intermediate values ([1,10^2^] min). Thus, the system is set up to maximally respond to rapid changes in the environment but not to the long day and night oscillations registered under physiological conditions. Interestingly, the model predicts a small range of logarithmic amplification values (*LA≈ *2), which means that the average amount of STAT5 units activated per activated receptor remains very similar (around one hundred units) for a wide interval of Epo concentrations.

The system acts as a strong amplifier with respect to the scale defined in the three kinds of processes simulated and analysed. The model predicts that on average one activated dimeric EpoR can provoke the activation and subsequent nuclear translocation of approximately one hundred molecules of STAT5. Another interesting property is that the system seems to be more efficient when weak stimuli are applied.

For each model structure analysed, we stored a large collection of solutions and then analysed their properties. We found that, although the fitting to the data was not completely satisfactory in some of these solutions, a significant part of them showed similar properties related to the responsiveness and amplification of the system. Both type of models analysed (fixed predefined integer versus variable non-integer kinetic orders) show the characteristic sigmoid response curve, but there is a significant difference between them when the population of the solutions is considered. For the model with fixed integer kinetic orders there is a little variation between the 100 best solutions. On the other hand, for the the power-law model with variable non-integer kinetic orders there is a variety of solutions that reproduce sigmoid response curves including those of the model (as shown in Sup. Mat. A8 [see Additional file 1]). We think that this property relates to the results shown in [29], where the authors suggested that key properties of the biochemical networks, including signalling pathways, should be at least partially robust (in the sense of not-requiring "fine-tuning" of parameters) in order to ensure their proper functioning. With regard to this idea, we think that the sigmoid response curve obtained for the JAK2-STAT5 pathway mostly relates to the structure of the pathway and not to the precise values of the model parameters. In this case, the JAK2/STAT5 could be considered as a robust amplifier.

The final conclusion is that the JAK2/STAT5 system acts as an amplifier of the signal, which has the maximum sensitivity for input signals whose intensity coincides approximately with the physiological values, and reaches saturation for very intense and long stimulation. The general concepts, definitions and strategy of analysis proposed here could in principle be used to analyse the properties of any pathway once the dynamics of their regulatory proteins are measured.

## Methods

### Experimental techniques

Phoenix-eco cells were transiently transfected with the retroviral expression vector pMOWS-HA-EpoR by calcium-phosphate as previously described [30]. Six hours after transfection, Phoenix-eco cells were cultured in Iscove's Modified Dulbecco's Medium (IMDM) (Invitrogen) containing 50 μM β-mercaptoethanol (Invitrogen) and 30% fetal calf serum (Invitrogen). Virus-containing supernatant was harvested 24 h after transfection and filtered (0.45-μm filter, Corning). For spin infection, 10^5 ^BaF3 cells were transduced with viral supernatants supplemented with 8 μg/ml Polybren (Sigma-Aldrich). BaF3 cells stably expressing HA-EpoR were selected in 1.5 μg/ml puromycin (Sigma-Aldrich) 48 hours after transduction.

For measuring activated Epo receptor and STAT5, HA-EpoR-BaF3 cells were starved in RPMI 1640 (Invitrogen) and 1 mg/ml BSA (Sigma-Aldrich) for 5 h. Cells were stimulated with 5 units/ml Epo (Janssen-Cilag) at 37°C for indicated times and 10^7 ^cells per time point were lysed with NP-40 lysis buffer (1% NP-40, 150 mM NaCl, 20 mM Tris pH7.4, 10 mM NaF, 1 mM EDTA pH 8.0, 1 mM ZnCl_2 _pH4.0, 1 mM MgCl_2_, 1 mM Na_3_VO_4_, 10 % glycerol) supplemented with aprotinin and AEBSF (Sigma-Aldrich). For immunoprecipitation, lysates were incubated with anti-EpoR antibodies (Santa Cruz) and anti-STAT5 antibodies (Santa Cruz). 40 ng of GST-EpoR and 36 ng GST-STAT5b was added as calibrator to each lysate as described [4]. Immunoprecipitated proteins were loaded in randomised fashion on SDS polyacrylamide gel as described [4], separated by electrophoresis and immunoblotted using anti-phosphotyrosine monoclonal antibody 4G10 (Upstate Biotechnology) and secondary HRP coupled anti-mouse antibody (Amersham Biosciences). Immunoblots were incubated with ECL substrate (Amersham Biosciences) for 1 min and exposed for 10 min on a LumiImager (Roche Diagnostics). LumiAnalyst software (Roche Diagnostics) was used for quantification. Antibodies were removed by treating the blots with β-mercaptoethanol (Sigma-Aldrich) and SDS (Serva) as described [31]. Reprobes were performed using anti-EpoR antibody (Santa Cruz) and anti-STAT5 antibody (Santa Cruz). Quantitative immunoblotting data was processed using GelInspector software [3]. Normalisers GST-EpoR for pEpoR and GST-STAT5b for pSTAT5 were used. For first estimates, csaps-splines were used with a smoothness of 0.3 for pEpoR and pSTAT5.

For measuring the extracellular Epo, an independent experiment was performed in which BaF3 cells stably expressing murine EpoR were starved in RPMI 1640 (Invitrogen) supplemented with 1 mg/ml BSA (Sigma-Aldrich) for 3 h and subsequently stimulated for up to 180 min with 5 units/ml [^125^I]-Epo (Amersham Biosciences) at a density of 4 × 10^6 ^cells/100 μl medium and 37°C. To separate free [^125^I]-Epo from cell-associated [^125^I]-Epo, cells were centrifuged through a layer of fetal calf serum (Invitrogen) and supernatants were measured in a gamma counter (Packard). Measurements were performed in triplicates. As control, cells were additionally incubated with an excess of unlabeled Epo (100 units/ml) (Janssen-Cilag), showing no decrease in free [^125^I]-Epo in the medium.

### Mathematical modelling

The JAK2-STAT5 pathway has been modelled using a power-law representation [15]. As we discussed in Background, power-law models allow the representation of complex dynamics such as saturation-like behaviour, inhibition or cooperativity with simplified equations. Our strategy is to iteratively increase the complexity of our model by allowing variable non-integer kinetic orders for those processes where we do not have prior knowledge to determine the value of the kinetic order. We thus allow for a larger class of models, which includes the structure of conventional models based on mass-action kinetics (with fixed predefined integer kinetic orders) as a special case.

### Parameter estimation

In the present paper a genetic algorithm was used for parameter estimation. The algorithm has been adapted and optimised for power-law models. In the estimation process, each element of the population of solutions represents a point in the parameter value space. The initial population of solutions is generated through a random exploration of the search space, which is defined using feasible intervals of values for the variables (Sup. Mat. A2 [see Additional file 1]). The best individuals of the population are selected in the considered iteration based on the value of the following objective function [32]:

(2) $$F_{obj}=\frac{1}{n_{\exp}\cdot n_{\mathrm{var}}\cdot n_{tp}}\sum_{k=1}^{n_{\exp}}\sum_{j=1}^{n_{\mathrm{var}}}\sum_{i=1}^{n_{tp}}{\left(X_{k,j}\left(t_{i}\right)-X_{k,j}^{\exp}\left(t_{i}\right)\right)}^{2}$$

where *n*_*exp *_is the number of experiments, *n*_*var *_is the number of measured quantities (observables), *n*_*tp *_the number of time points where each observable was measured, *X*_*k, j*_(*t*_*i*_) the value of the *j*^*th*^observable at the *i*^*th *^time point obtained after numerical integration of the solution for the *k*^*th *^experiment. $X_{k,j}^{\exp}\left(t_{i}\right)$ is the value of the *j*^*th *^observable at the *i*^*th *^time point measured in the *k*^*th *^experiment. An additional fast-climbing stochastic algorithm is applied to the best solution each iteration of the algorithm. The stopping criterion is based on either a previously established maximum number of iterations or a minimum level of satisfaction for the objective function. Computations were performed on a Sun Fire V880 Server (four processors UltraSPARC-III, 1200 MHz, 8 MB cache each; RAM memory 32 GB). The algorithm for parameter estimation was implemented in Matlab R14 (The Mathworks, Inc. Natick, MA) running under SunOS 5.10.

## Authors' contributions

JB, VB and ACP from the DKFZ (Heidelberg) carried out the experimental assays necessary to generate the data used in this work under the supervision of UK. JV from the University of Rostock designed the study, set up the mathematical model and performed the calculations concerning the analysis of responsiveness and amplification under the supervision of OW. JAH from University of La Laguna calibrated the model and generated the mathematical simulations used in this work under the supervision of NVTD. Finally all the authors including JT from University of Freiburg drafted the manuscript.

## Supplementary Material

Additional file 1 Supplementary material. The additional file contains further information about the model calibration process and some figures complementing the discussion and the model selection. Click here for file
