# Supplementary material for: A systems biology approach to analyse amplification in the JAK2-STAT5 signalling pathway
Source: BMC Syst Biol. 2008 Apr 25;2:38. doi: 10.1186/1752-0509-2-38 (PMC2386439; doi:10.1186/1752-0509-2-38)
Supplement: Additional file 1 — Supplementary material. The additional file contains further information about the model calibration process and some figures complementing the discussion and the model selection. [file 1752-0509-2-38-S1.pdf]

A systems biology approach to analyse amplification in the JAK2-STAT5 signalling pathway

Julio Vera[1] , Julie Bachmann[2], Andrea C. Pfeifer[2], Verena Becker[2], Jose A. Hormiga[3], Nestor V. Torres Darias[3], Jens Timmer[4], Ursula Klingmüller[2], and Olaf Wolkenhauer[1,*]

[1]Systems Biology and Bioinformatics Group, Department of Computer Science. University of Rostock. Rostock, Germany
[2]Systems Biology of Signal Transduction Group. German Cancer Research Center (DKFZ). Heidelberg, Germany
[3]Biochemical Technology Group, Department of Biochemistry and Molecular Biology. University of La Laguna. La Laguna, Spain
[4]Physics Institute, University of Freiburg, Freiburg, Germany

Supplementary Material

A1. Data processing (prior to parameter estimation) and additional hypothesis applied to the model

Variables in the model were normalised to avoid numeric problems during the parameter estimation. For the modelling, we assume that the total amount of EpoR at the plasma membrane after starvation and before the experiment is equal to one while the total amount of STAT5 is assumed to be constant and equal to one during the experiment. Therefore, values for system variables were chosen between zero (no proteins in the considered state) and one (total available amount of protein in this state). Since there is no complete balance in the equations of the model between degradation, deactivation and recruitment of the receptor complex, the total amount of the EpoR at the cell surface can vary during the experiment. In case of STAT5 and after considering that the variables *DpS* and DpS_{nc} are dimers, the equation describing this mass conservation is the following:

$$S + 2 \cdot DpS + 2 \cdot DpS_{nc} = STAT_{tot} = 1$$

In the normalised scale used in the model, the concentration of Epo in the extracellular medium at the starting point of the experiment (Epo=5 unit/ml) was also considered equal to one.

STAT5 activation is composed by two consecutive processes: monomer phosphorylation (leading to *pS*) and subsequent dimerisation of phosphorylated monomers (leading to *DpS*):

$$pEpJ + S \rightarrow pS \text{ (slow)}$$

$$pS + pS \rightarrow DpS \text{ (fast)}$$

The dynamics of *pS* and *DpS* can be described with the following differential equations:

$$\frac{d}{dt} pS = \gamma' \cdot S^{g_6} \cdot pEpJ^{g_7} - 2\gamma'' \cdot pS^2$$

$$\frac{d}{dt} DpS = \gamma'' \cdot S^2 - \gamma'' \cdot pS^{g'}$$

Preliminary results suggested that dimerisation is a fast processs and therefore the slower phosphorylation of STAT5 in the receptor complex leads the activation process. Under this assumption, the fraction of monomeric phosphorylated STAT5 can be neglected and the dynamics of

* **Corresponding author:** Olaf Wolkenhauer. Systems Biology and Bioinformatics Group. Department of Computer Science, University of Rostock. 18051 Rostock, Germany.
E-mail: olaf.wolkenhauer@uni-rostock.de Web: www.sbi.uni-rostock.de
Tel.: +49 381 498 75 70 Fax: +49 381 498 75 72

the activation process can be reduced to the following differential equation, which accounts for the active dimerised STAT5, *DpS*:

$$\frac{d}{dt} pS = \gamma' \cdot S^{g_6} \cdot pEpJ^{g_7} - 2\gamma'' \cdot pS^2 \approx 0 \Rightarrow \gamma' \cdot S^{g_6} \cdot pEpJ^{g_7} = 2\gamma'' \cdot pS^2$$

$$\Rightarrow \frac{d}{dt} DpS = 1/2 \cdot \gamma' \cdot S^{g_6} \cdot pEpJ^{g_7} - \gamma'' \cdot pS^{g'} \Rightarrow \frac{d}{dt} DpS = \gamma_5 \cdot S^{g_6} \cdot pEpJ^{g_7} - \gamma_6 \cdot DpS^{g_8}$$

Data from two replicate experiments were available and used for parameter estimation. The cytoplasmic concentration of activated STAT5, [$pSTAT5_{cyt}$], and the concentration of activated EpoR/JAK2 complex, [pEpoR], were measured in several timepoints. Additionally, the extracellular concentration of Epo, [Epo], was measured in an independent equivalent experiment. Additional algebraic equations, reflecting the relation between the measured quantities (observables) and the variables, were defined in the model:

[Epo] = *Epo* [pEpoR] = *pEpJ* [$pSTAT5_{cyt}$] = $2 \cdot DpS$

The variables on the left hand-side represent the observables, while the variables on the right-hand side represent the variables considered in the model. We finally have appropriately scaled the data. Additional biological assumptions were used to establish the proportion of protein activated in the peaks of stimulation for both variables *pEpJ* and *DpS*. Dose-response experiments performed with BaF3-HA-EpoR cells showed saturation for Epo binding to EpoR for concentrations of stimulus around the value (5 unit/ml Epo) used in the experiment (data not shown). Thus, we assume for our calculations that almost the total available amount of EpoR at the plasma membrane (95%) is bound to Epo and subsequently activated at the peak of activation. Results obtained from immunoblotting (data not shown) supported the assumption that 60% of the total amount of STAT5 in the cell was in the cytosol at the peak of activation. Furthermore, it can be assumed that approximately 60% of the cytosolic STAT5 were activated and dimerised in the peak of activation, which means that 36% of the total amount of the protein was activated, cytosolic STAT5 at the peak of activation. Once the proportion of the activated EpoR and STAT5 in the peaks was known, the remaining data were adequately scaled according to these values to obtain the data shown in Figure 2 of the main text of the article. The initial state of the system, after starvation and before stimulation, was approximated by assuming that virtually the entire amount of EpoR/JAK2 complex at the cell surface (99%) and STAT5 (98%) was in an inactive state. This permits the assignment of fixed initial conditions to the variables:

Epo(0) = 1.00 *EJ*(0) = 0.99 *pEpJ*(0) = 0.01

S(0) = 0.98 *DpS*(0) = 0.005 $DpS_{nc}(0)$ = 0.005

Both experimental data and initial conditions were used to estimate parameter values.

The data for extracellular Epo were used as input signal of the system. The initial concentration of Epo was known, and then measured during the experiment. These data were used to estimate a spline of the time-dependent Epo concentration, which was used as input signal of the system for the parameter estimation process (see Figure A1.1).

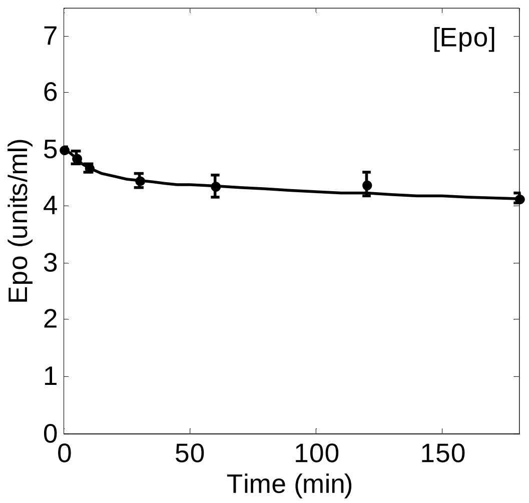

Figure A1.1. Experimental data obtained (black points with error bars) and the subsequently generated spline for the time-dependent concentration of Epo (black solid line). The concentration of Epo in the medium was measured by incubating BaF3-EpoR cells with [^{125}I]-Epo approximately equal to 5 units/ml Epo.

A2. Model selection, parameter estimation and data fitting

A strategy for model selection was used to decide on the most suitable structure for the model with a reduced number of parameters. The idea was to obtain the simplest possible mathematical model, which encoded the biological knowledge available and allowed for an acceptable prediction of experimental data. The strategy used was based on three criteria:

i) We only considered non-integer kinetic orders for the EpoR/JAK2 complex activation and the STAT5 activation rates, which we know are encoding underlying complex mechanisms.
ii) We did not consider all selected kinetic orders to be non-integer at the same time, but defined an iterative process in which an increasing number of kinetic orders were allowed to be variable and different to one.
iii) Finally, we selected the first (simplest) model that allows an appropriate fit to experimental data with no significant improvement in the next (more complex) model formulated and tested (see Table A2.3 for results of this comparison).

The parameter defining the time delay of STAT5 in the nucleus was analysed in a similar way and models with and without time delay were considered and computed. In all cases, parameters were computed for biologically feasible intervals in the parameter space (Table A2.1). In the normalised scale of the variables used the interval used for parameters γ_2-γ_6 allow for the representation of processes which characteristic timing ranges between approx. 1 minute (which is the time resolution of the experiments used), upper bound γ=0.5 and 150 minutes (which is approx. the slowest process that could be measured in the time interval of the experiment), lower bound γ=0.005. In case of γ_1, a specific range was established which represents the half-life of non-activated receptor between 0.1 and 20 times the values described in Supino-Rosin et al. (1999)[1]. The feasible values for kinetic orders (g_1-g_8) range between strong saturation (lower bound for g around 0.25) and significant cooperativity (upper bound for g around 1.75). Finally, we assumed that the value of the time delay (τ) computed in Swameye et al. (2003) for almost identical cells is valid for our experiments and therefore allowed an

[1] Supino-Rosin L, Yoshimura A, Altaratz H, Neumann D. (1999) A cytosolic domain of the erythropoietin receptor contributes to endoplasmic reticulum-associated degradation. Eur J Biochem. 1999 Jul;263(2):410-9.

interval of values for τ between 0.5 and five times the previously established value in Swameye et al. (2003). The time delay was modelled using the linear chain trick (size two) described in McDonald (1978)[2]. Parameter estimation was performed with a hybrid method composed of a genetic algorithm for the global search and an additional fast-climbing stochastic algorithm, applied to the best solution in each iteration. The stopping criterion is based on a previously established maximum number of iterations (in the case analysed, 250 iterations).

Table A2.1. Feasible intervals of parameter values

**Parameter**	g_1-g_8	γ_1	γ_2	γ_3	γ_4	γ_5	γ_6	τ
Upper Bound	1.75	0.003	0.5	0.5	0.5	0.5	0.5	35
Lower Bound	0.25	0.00001	0.005	0.005	0.005	0.005	0.005	3.5

Table A2.2. Model structures tested

**Model tested**	**Conditions required in parameters**
Conventional kinetic model	$g_1=g_2=g_3=g_4=1$ and $\tau=0$
Conventional kinetic model with time delay	$g_1=g_2=g_3=g_4=1$ and $\tau>0$
Complexity in receptor activation	$g1\neq1$ and $g2\neq1$ and $g3=g4=1$ and $\tau=0$
Complexity in STAT5 activation	$g1=g2=1$ and $g3\neq1$ and $g4\neq1$ and $\tau=0$
Complexity in STAT5 activation with time delay	$g1=g2=1$ and $g3\neq1$ and $g4\neq1$ and $\tau>0$

Table A2.3. Comparison of the performance of different models analysed.

**Model tested**	F_{obj}	% F_{obj}[3]	Nº parameters
Conventional kinetic model	0.0617	+20%	6
Conventional kinetic model with time delay	0.0896	+75%	7
Complexity in receptor activation	0.0513	0%	8
Complexity in STAT5 activation	0.0912	+78%	8
Complexity in STAT5 activation with time delay	0.0739	+44%	9

We focus our discussion on the model with fixed predefined integer kinetic orders and the one with variable non-integer kinetic orders in the term representing the receptor activation, which represent clearly two different options with respect to the simplicity and accuracy of the model. The model with variable non-integer kinetic orders fits the data better (the objective function, F_{obj}, is 20% smaller) but has a higher proportion of additional parameters when compared with the other model. As shown in Figure A2.1, the improvement in the data fitting by the model with variable non-integer kinetic orders is not perceptible and both solutions are almost identical and are probably in the limit of accuracy of the provided experimental data. Furthermore, if we compare the dynamical simulations provided for both models for the fraction of activated STAT5 in the nucleus and the fraction of inactive cytosolic STAT5 (Figure A2.2), differences are also not significant. By applying the parsimony principle, we decided to choose the model with fixed predefined integer kinetic orders as an appropriate description of the system and used it in our analysis. However, in the last sections of the Supplementary Material

[2] MacDonald N. 1978. Time Lags in Biological Models. Springer, Heidelberg (1978).

[3] $\%F_{obj}$ measures the difference in the value of the objective function between the considered solution and the best one in terms of data fit (complexity in receptor activation): $\%F_{obj} = \frac{F_{obj} - 0.0513}{0.0513}$

(A7 and A8) we have included a comparison between both models for the properties analysed in our investigation.

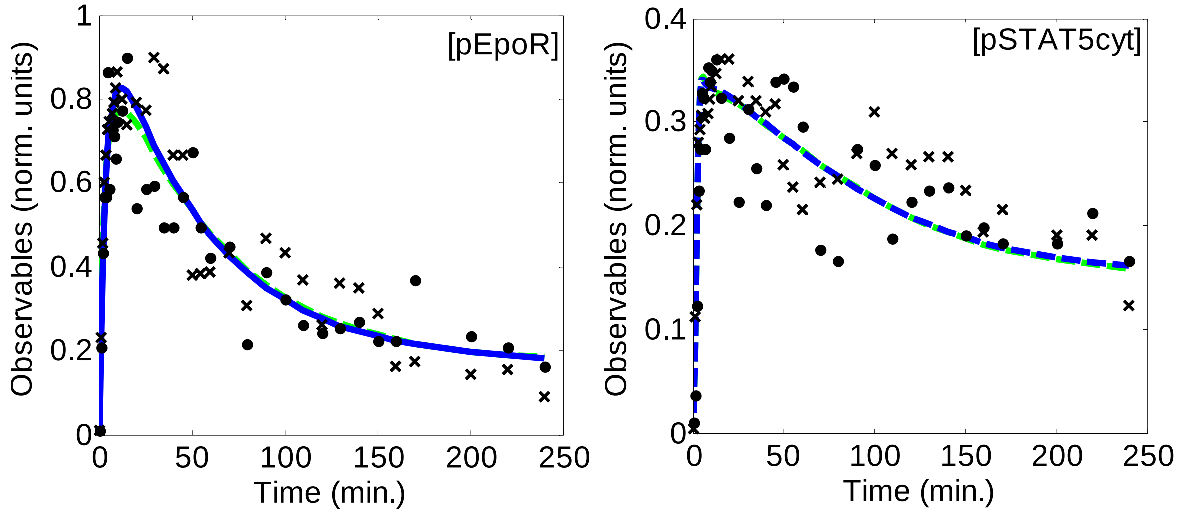

Figure A2.1. Comparison of the data fitting for the model with fixed predefined integer kinetic orders (solid blue line) and the model with variable non-integer kinetic orders in the representing the receptor activation (dashed green line).

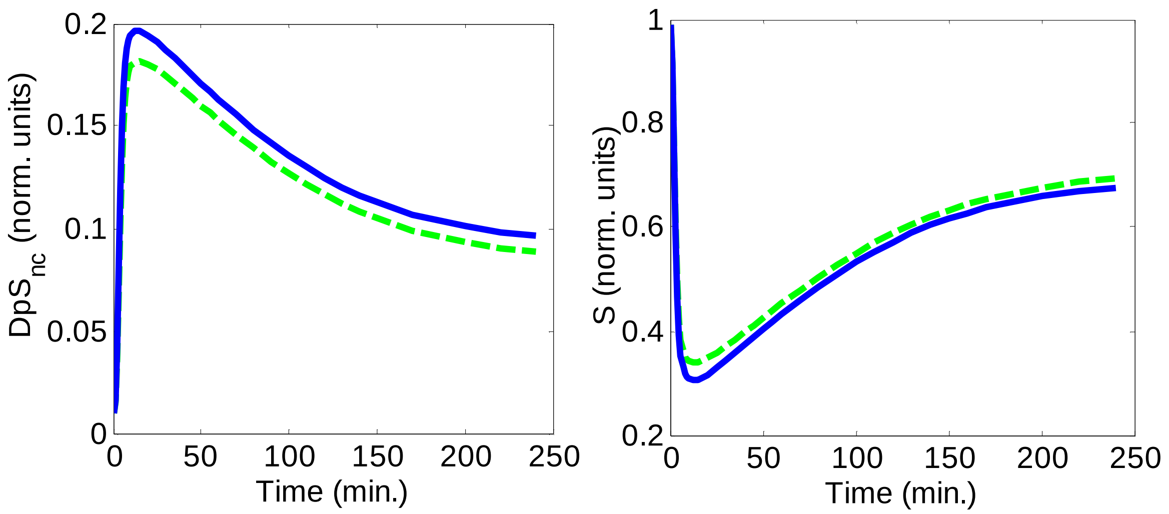

Figure A2.2. Comparison of the simulations for the dynamics of nuclear activated STAT5 (DpSnc, left) and cytosolic monomeric STAT5 (S, right) during the model with fixed predefined integer kinetic orders (solid blue line) and the model with variable non-integer kinetic orders in the representing the receptor activation (dashed green line**.**

Figure A.2.3 shows an extended simulation of the dynamics of the system after perturbation with Epo as used in the experiments for the solution selected. The majority of the EpoR/JAK2 complex which is initially at the plasma membrane is activated very quickly (approx. 85% after 20 min.). However, the concentration of *pEpJ* goes down very fast after the peak of activation due to the effects of intense deactivation. The amount of activated STAT5 in the cytosol (*DpS*) increases very quickly after stimulation and reaches the maximum around 15 minutes after stimulation. Afterwards, it maintains a high value even if the value of activated receptor *pEpJ* decreases by 80% at the end of the experiment. The increase in the fraction of activated STAT5 in the nucleus (DpS_{nc}) after stimulation is slightly delayed.

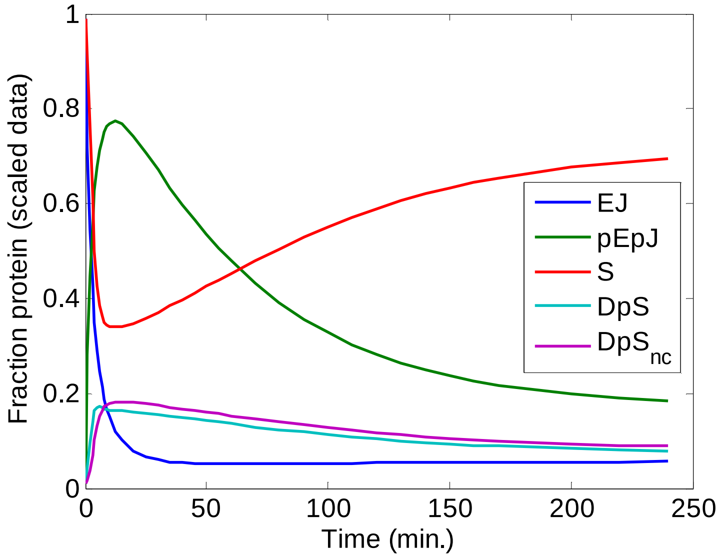

Figure A2.3. Simulation of the dynamics of system after perturbation including all the variables of the model. Legend: *EJ*: inactive EpoR/JAK2 complex at he plasma membrane; *pEpJ*: activated EpoR/JAK2 at the plasma membrane; S: inactive monomeric STAT5 in the cytosol; *DpS*: activated dimeric STAT5 in the cytosol; DpS_{nc}: activated dimeric STAT5 in the nucleus.

A3. Consequences of logarithmic properties on the definition of the logarithmic amplification

The properties of logarithmic functions allow us to separate the ratio between both proteins in the definition of *LA* in the pathway studied:

$$LA = \log\left(R_{STAT5/EJ} \frac{\int_0^T V^{+}_{DpS_{nc}}(t)\cdot dt}{\int_0^T V^{+}_{pEpJ}(t)\cdot dt} \right) = \log\left(R_{STAT5/EJ}\right) + \log\left(\frac{\int_0^T V^{+}_{DpS_{nc}}(t)\cdot dt}{\int_0^T V^{+}_{pEpJ}(t)\cdot dt} \right)$$

Since the ratio between the total amount of both proteins is considered stable during the experiments performed, the logarithm of $R_{STAT//EJ}$ is a constant for the analysis we are performing. This means that in the graphical representation of *LA*, versus the stimulus, *Epo*, the value of this ratio translates the position of the curve but does not alter other properties of the figure such as scale, shape and position of maximum and minimum. Changes in the value of this ratio would appear only in case of considering experiments or processes in which the ratio between both proteins can be modulated.

A4. Analysis of transient stimulus

We were interested in investigating the behaviour of the system with transient signals with a pulse-like structure. Towards this end, we designed simulated transient perturbations with a shape as described in Figure A5.1.

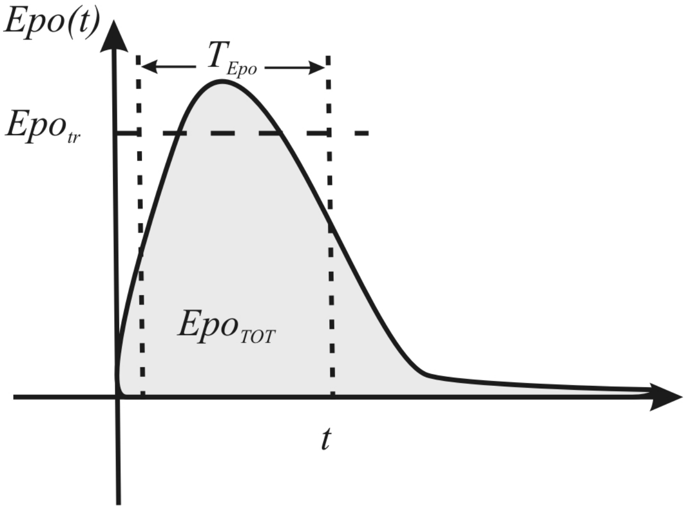

Figure A4.1. Design of a typical transient stimulus used in our analysis. The properties characterising the signal are average duration, T_{Epo}, and average value of stimulus, Epo_{tr}.

The magnitudes which characterise the simulated transient stimulation were adopted from Heinrich et al. (2002). Hence, the duration of the signal is described by T_{Epo}, which is the average duration of the signal and is defined by the following equation:

$$T_{Epo} = 2\sqrt{\frac{\int_o^{\infty} \left(t - \langle\tau\rangle\right)^2 Epo(t)\cdot dt}{Epo_{TOT}}}$$

where:

$$Epo_{TOT} = \int_{t=0}^{t=\infty} Epo(t) \cdot dt \qquad \langle \tau \rangle = \frac{1}{Epo_{TOT}} \int_{0}^{\infty} t \cdot Epo(t) \cdot dt$$

The other variables that characterise the simulated transient stimulus is Epo_{tr}, which accounts for the intensity of the signal and represents the average value of Epo during the transient stimulus. This variable is defined as:

$$Epo_{tr} = \frac{Epo_{TOT}}{T_{Epo}}$$

In a similar manner, the average intensity of the signal for the activated receptor, *pEpJ,* and the fraction of dimeric activated STAT5 in the nucleus, DpS_{nc}, are defined with identical equations:

$$pEpJ_{tr} = \frac{\int_{t=0}^{t=\infty} pEpJ(t) \cdot dt}{T_{pE}} \qquad DpS_{nc,tr} = \frac{\int_{t=0}^{t=\infty} DpS_{nc}(t) \cdot dt}{T_{DpS_{nc}}}$$

where T_{pEpJ} and T_{DpSnc} are representing the average duration of the signal for *pEpJ* and DpS_n and are estimated using the same equations used in case of T_{Epo}. We calculated the defined magnitudes by numerical integration. In all cases, the interval of numerical integration was 5.000 minutes for simulations with transient stimulation smaller than $T_{Epo}=10^3$ (that is, integration time was ten to thousands of times longer than input signal duration). In case of longer transient stimulation, results with longer integration time did not differ to the results with 5.000 minutes, which relates to the importance of low Epo receptor recruitment when long intense stimulation is applied (see discussion in the main text).

A5. Oscillatory stimulus analysis

The oscillatory perturbation applied to the system is described by Figure A6.1.

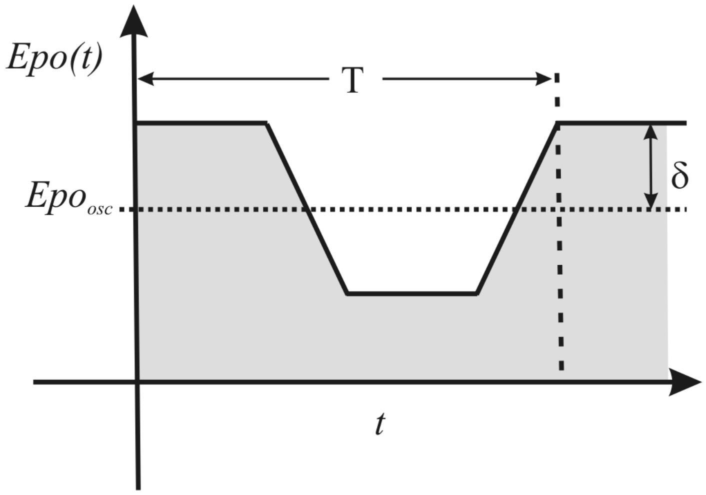

Figure A5.1. Design of a typical oscillatory stimulus used in our analysis, which is proposed to be a series of truncated triangular peaks. The properties characterising the signal are the period of oscillation in the stimulation, T, the amplitude of the oscillation, δ, and the average value of stimulus, Epo_{osc}.

During the visual inspection of the simulation we did not discover the emergence of new oscillations in any of variables with a period different to the one assigned to the oscillation in Epo. The oscillations provoked a signal with the same period for the all the variables (*pEpJ*, *DpS* and DpS_{nuc}) but displaced on time (representing the time that the signal takes to reach the different steps in the pathway). In order to avoid the problems associated with this displacement in the different variables,

the following magnitudes were estimated for 12 consecutive periods and then the values were averaged. Epo_{osc} is the average value of Epo during the oscillatory stimulus and is defined by:

$$Epo_{osc} = \frac{\int_{t=0}^{t=t_f} Epo(t) \cdot dt}{T}$$

where T is the period of the simulated oscillatory stimulus. Average values of *pEpJ* ($pEpJ_{osc}$) and DpS_{nc} ($DpS_{nc,osc}$) during oscillation are defined by the following equations:

$$pEpJ_{osc} = \frac{\int_{t=0}^{t=t_f} pEpJ(t) \cdot dt}{T} \qquad DpS_{nc,osc} = \frac{\int_{t=0}^{t=t_f} DpS_{nc}(t) \cdot dt}{T}$$

A6. Figures showing the behaviour of *pEpJ* for different kinds of stimulus in the selected kinetic model (IKO)

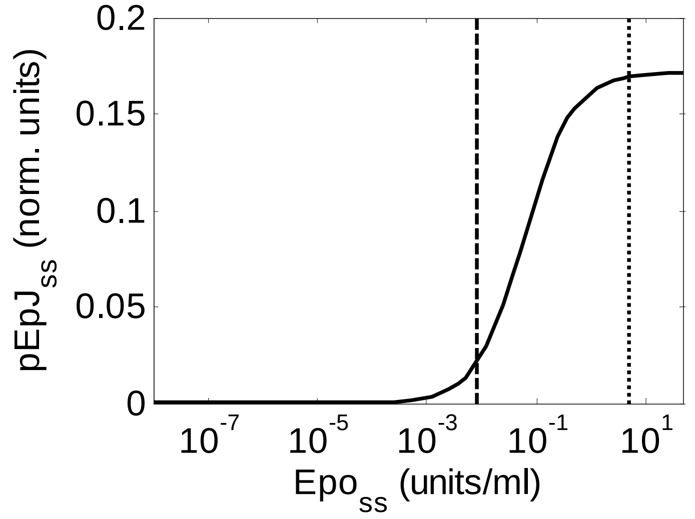

Figure A6.1. Steady-state values of activated receptor, *pEpJ* (norm. units), for different values of sustained stimulation on Epo (Epo_{ss}) (units/ml) in case of the chosen kinetic model (IKO). The dashed black line indicates the physiological value in mouse (7.9 mU/ml).

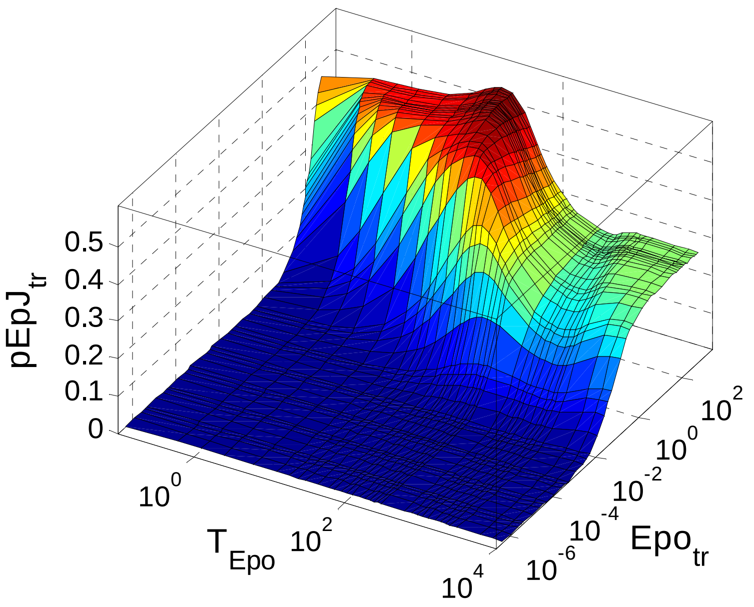

Figure A6.2. Average fraction of activated receptor $pEpJ_{tr}$ (norm. units), during transient stimulation in case of the chosen kinetic model (IKO). The behaviour of the system was analysed for transient stimulation with an average duration of $T_{Epo} \in [0.1, 10^4]$ minutes, and an average concentration of $Epo_{tr} \in [10^{-6}, 10^2]$ units/ml.

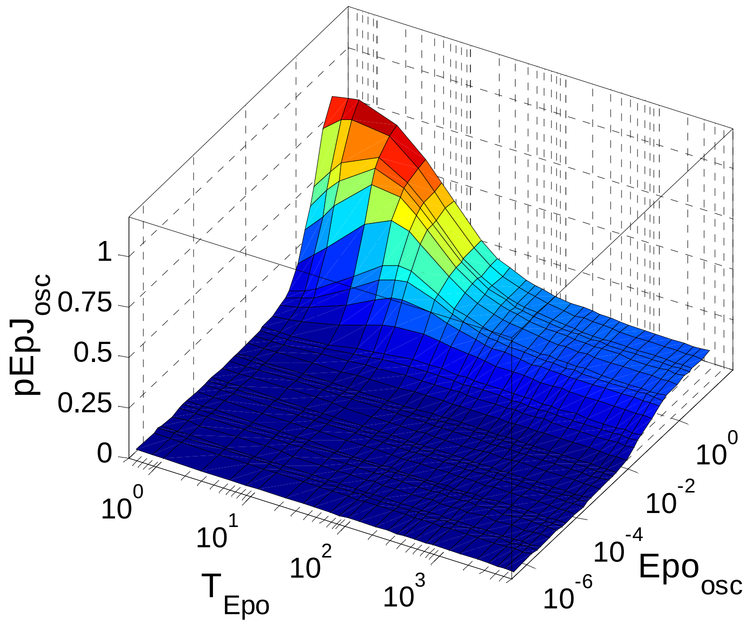

Figure A6.3. Average fraction of activated receptor, $pEpJ_{osc}$ (norm. units), during oscillatory stimulation in case of the chosen kinetic model (IKO).. The simulated data was averaged for several following periods. The behaviour of the system was analysed for oscillatory stimuli with an average period duration $T \in [0.5, 1440]$ minutes, and an average concentration of Epo_{tr} $[10^{-6}, 10]$ units/ml.

A7. Responsiveness in the model with fixed predefined integer kinetic orders (IKO model) and the model with variable non-integer kinetic orders (NIKO model)[4]

A7.1 Sustained stimulation

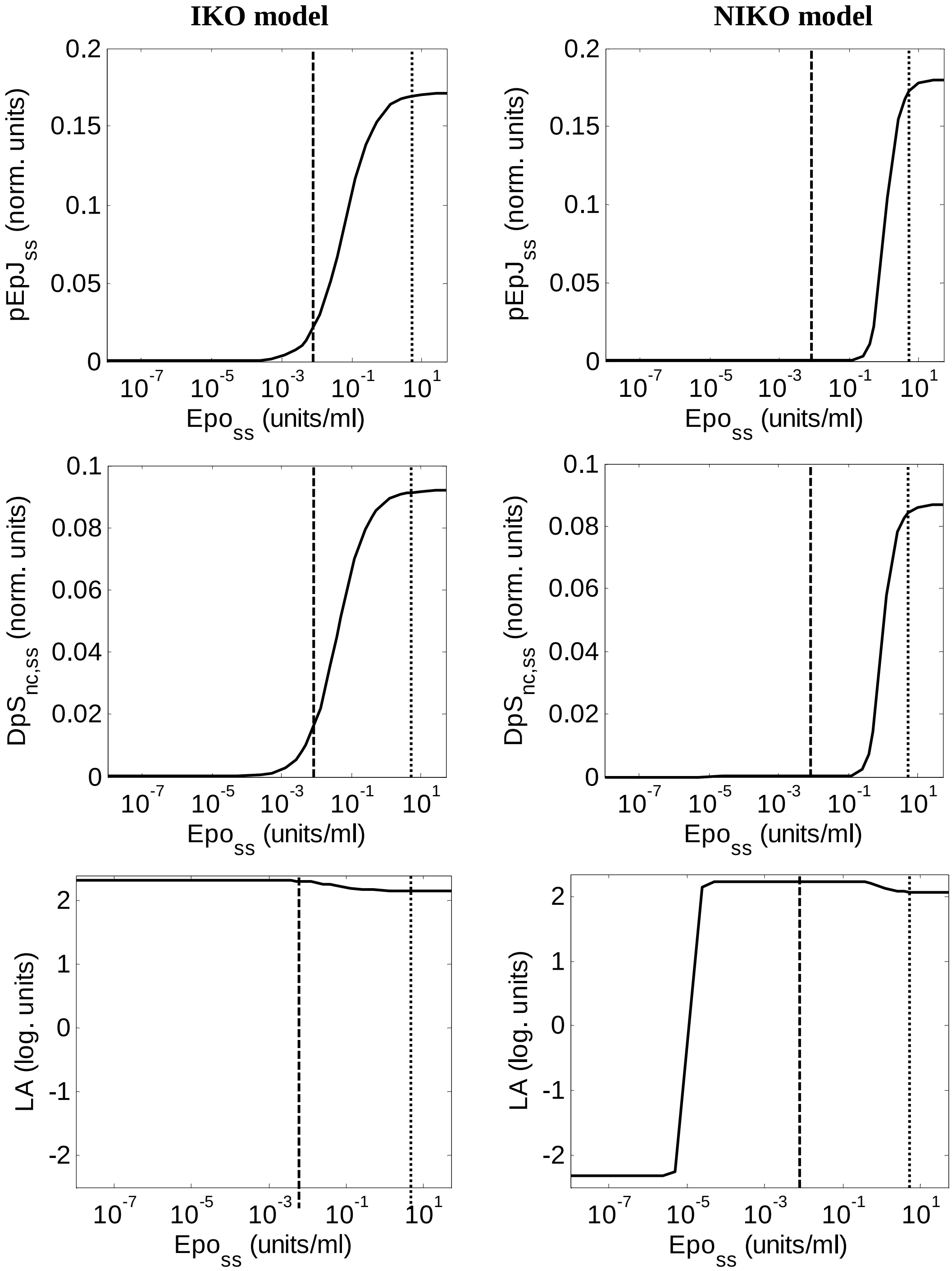

Figure A7.1. Comparison between the model with fixed predefined integer kinetic orders (IKO model, left) and the model with variable non-integer kinetic orders (NIKO model, right) versions of the model for the steady-state values of activated receptor (*$pEpJ_{ss}$*, top), activated STAT5 in the nucleus (*$DpS_{nc,ss}$*, middle) and logarithmic amplification (*LA*, bottom) under different values of sustained stimulation (*Epo_{ss}* ∈ $[10^{-9},10]$ units/ml).

[4] For a complete discussion of these figures, see final paragraph in Results and Discussion Section.

A7.2 Transient stimulation

IKO model **NIKO model**

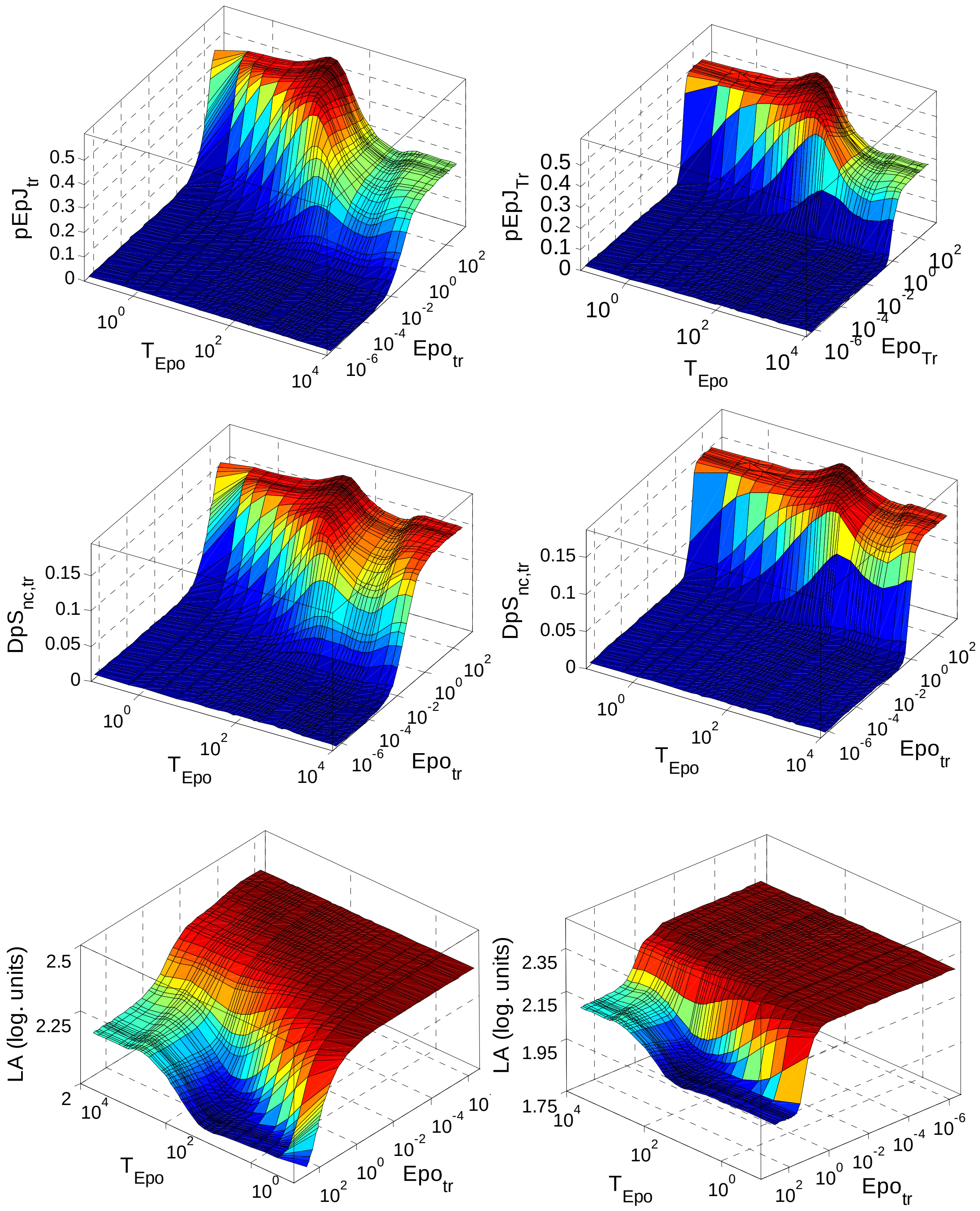

Figure A7.2. Comparison between the IKO and NIKO versions of the model for the average values of activated receptor (*pEpJ*$_{tr}$, top), activated STAT5 in the nucleus *(DpS*$_{nc,tr}$, middle) and logarithmic amplification *(LA*, bottom) under different values of transient stimulation ($T_{Epo} \in [0.1, 10^4]$ minutes, $Epo_{tr} \in [10^{-6},500]$ units/ml).

A7.3 Oscillatory stimulation

IKO model **NIKO model**

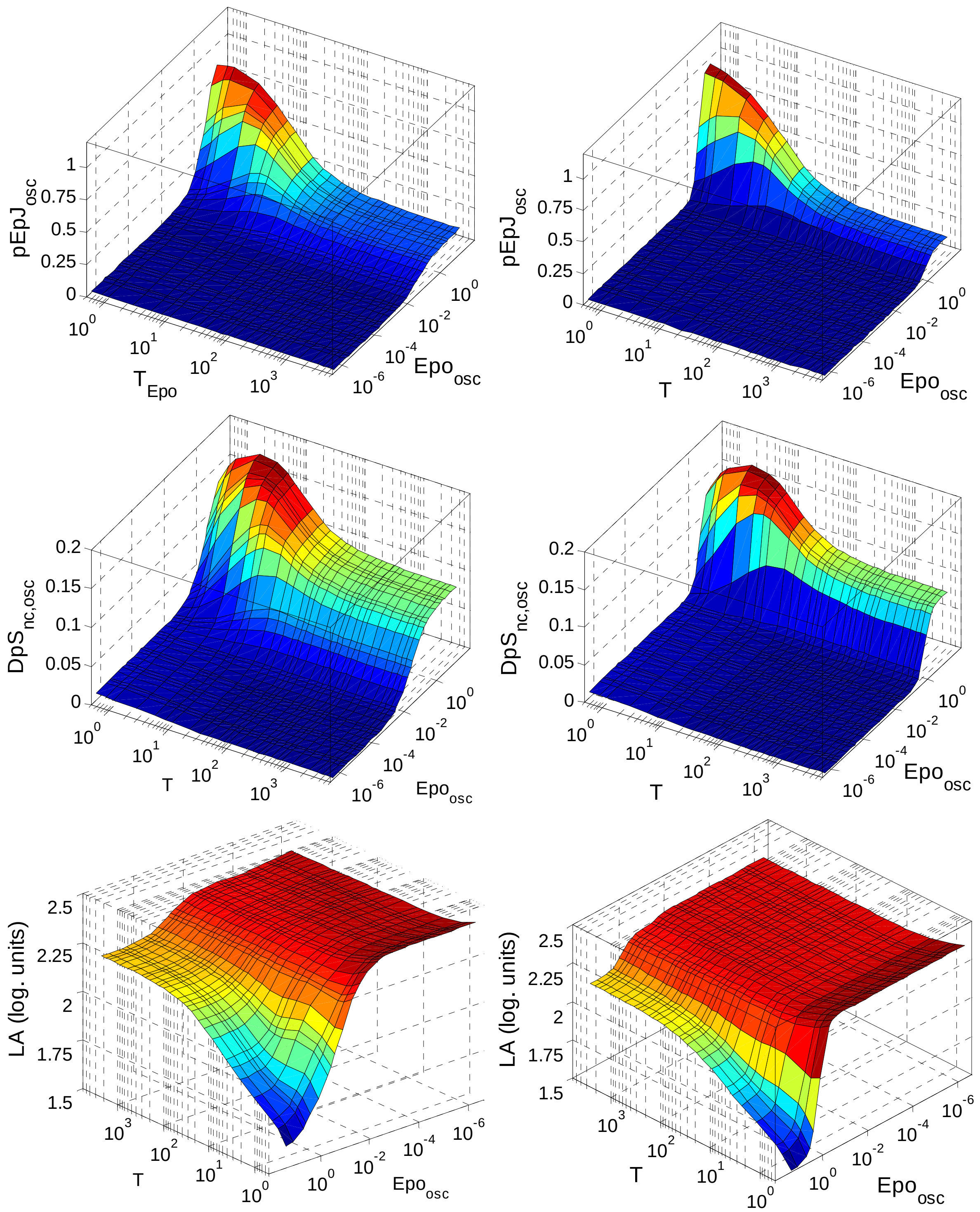

Figure A7.3. Comparison between the IKO and NIKO versions of the model for the average values of activated receptor ($pEpJ_{osc}$, top), activated STAT5 in the nucleus ($DpS_{nc,osc}$, middle) and logarithmic amplification (LA, bottom) under different values of oscillatory stimulation (period of oscillatory signal $T \in [0.5, 1440]$ minutes; average concentration of $Epo_{osc} \in [10^{-6}, 10]$ units/ml).

A8. Comparison in the responsiveness of the system for the population of the best solutions of the IKO and NIKO models

IKO model

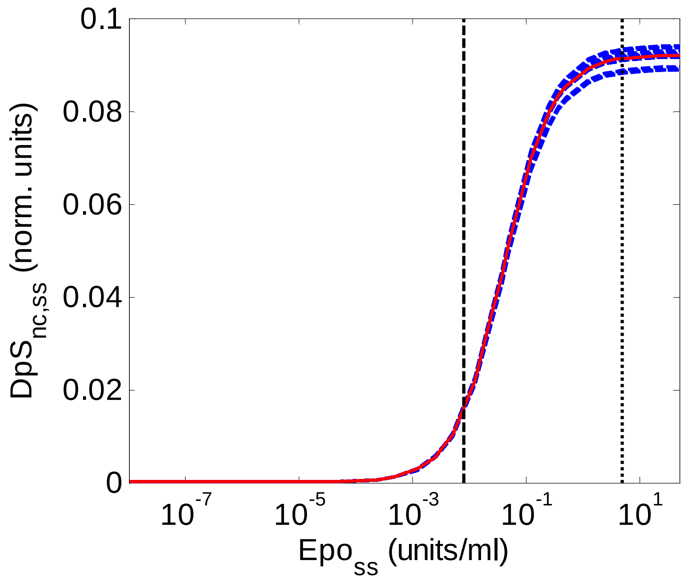

NIKO model

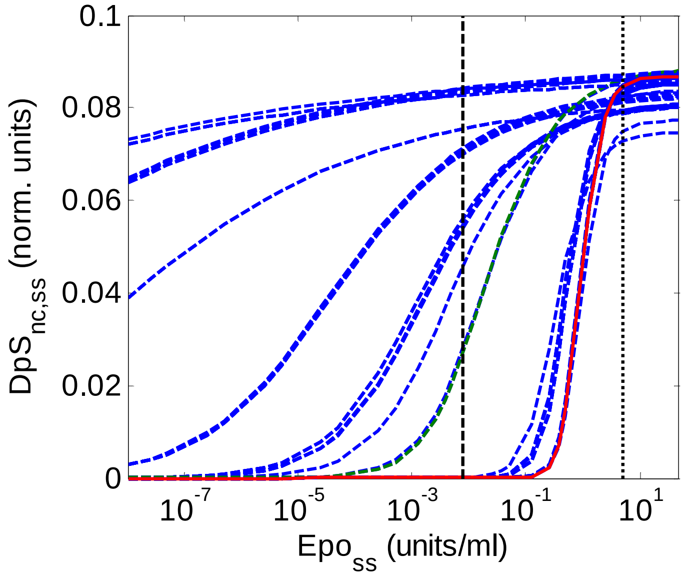

Figure A8. Comparison between the IKO (top) and NIKO (bottom) versions of the model for the steady-state values of activated STAT5 in the nucleus *($DpS_{nc,ss}$)* under sustained stimulation ($Epo_{ss} \in [10^{-9}, 10]$ units/ml) when not only the best solution but the population of the selected solutions is considered. In each figure, the behaviour of the selected solution appears as a red solid line and the other good solutions computed (100 first solutions) as a dashed blue line. In addition, for the model with variable non-integer kinetic orders (NIKO model) we marked in dashed green lines the solutions whose behaviour is most similar to the one encoded by the IKO solution.
